# Assessing β-Sitosterol Levels in Dietary Supplements for Benign Prostatic Hyperplasia: Implications for Therapeutic Efficacy

**DOI:** 10.7759/cureus.60309

**Published:** 2024-05-14

**Authors:** Buț Mădălina-Georgiana, Silvia Imre, Camil Vari, Bianca Eugenia Ősz, Ruxandra Ștefănescu, Amalia Pușcaș, George Jîtcă, Camelia-Maria Matei, Amelia Tero-Vescan

**Affiliations:** 1 Department of Medical Chemistry and Biochemistry, Faculty of Medicine in English, George Emil Palade University of Medicine, Pharmacy, Science, and Technology of Târgu Mureș, Târgu Mureș, ROU; 2 Doctoral School of Medicine and Pharmacy, George Emil Palade University of Medicine, Pharmacy, Science, and Technology of Târgu Mureș, Târgu Mureș, ROU; 3 Department of Analytical Chemistry and Drug Analysis, Faculty of Pharmacy, George Emil Palade University of Medicine, Pharmacy, Science, and Technology of Târgu Mureș, Targu Mures, ROU; 4 Department of Pharmacology and Clinical Pharmacy, Faculty of Pharmacy, George Emil Palade University of Medicine, Pharmacy, Science, and Technology of Târgu Mureș, Târgu Mureș, ROU; 5 Department of Pharmacognosy and Phytotherapy, Faculty of Pharmacy, George Emil Palade University of Medicine, Pharmacy, Science, and Technology of Târgu Mureș, Târgu Mureș, ROU; 6 Department of Biochemistry and Chemistry of the Environmental Factors, Faculty of Pharmacy, George Emil Palade University of Medicine, Pharmacy, Science, and Technology of Târgu Mureș, Târgu Mureș, ROU; 7 Department of Analytical Chemistry and Drug Analysis, Faculty of Pharmacy, George Emil Palade University of Medicine, Pharmacy, Science, and Technology of Târgu Mureș, Târgu Mureș, ROU

**Keywords:** plant-based compound, hplc-uv, dietary supplements, benign prostatic hyperplasia, β-sitosterol

## Abstract

Introduction

Benign prostatic hyperplasia (BPH) is a prevalent condition among aging men that affects their life quality due to urinary symptoms. Current pharmacologic treatments, often lead to sexual dysfunction, so dietary supplements (DS) containing plant-based compounds such as β-sitosterol (SIT) are preferred. DS are highly accessible and widely used, but poorly regulated, so often patients are victims of fraud. The use of DS to treat BPH symptoms is questionable, and this may be due not to the efficacity of the active compound but to the quality of commonly available DS.

Aim

This study aimed to assess the concentration of SIT in DS available on the market and evaluate whether the concentration of the active compound at the recommended dosage is sufficient to elicit beneficial effects in BPH.

Method

An HPLC-UV method based on direct saponification and acid hydrolysis was developed for the quantification of free and conjugated SIT in DS. The concentration of SIT in various DS was determined and compared with the one declared on the label.

Results

The chromatographic analysis confirmed the presence of SIT in all the DS but also showed a considerable variability of SIT content among DS, with only one product meeting the necessary concentration to bring potential benefits in BPH.

Conclusion

The study highlights inconsistencies in SIT content among DS and the importance of DS containing a standardized extract of SIT. Quality control measures are imperative to ensure that consumers receive effective and safe SIT-based DS to manage BPH symptoms. Further research is needed to establish standardized dosages and to evaluate their long-term efficacy and safety.

## Introduction

Benign prostatic hyperplasia (BPH), also known as prostate gland enlargement, is one of the most common conditions affecting men after a certain age. By the age of 60, more than half of men have some degree of BPH, and by the age of 85, the prevalence increases to approximately 90% [1].

Although it is considered a benign condition, it can still cause significant discomfort and affect the quality of life due to urinary symptoms such as frequent urination, urge incontinence, weak urine stream, and difficulty in starting or stopping urination [1]. Without being an emergency, BPH often requires medical attention and management, especially if symptoms become bothersome or significantly impact daily activities. Treatment options range from lifestyle changes and medications to surgical interventions, depending on the severity of symptoms and the individual's overall health [2]. When considering medical treatment exclusively, it can either address the symptoms or target the root cause, dihydrotestosterone (DHT). DHT is the active metabolite of testosterone [3]. Elevated activity of 5α-reductase, the enzyme responsible for converting testosterone to DHT, or increased activity of androgen receptors within the prostate, have been identified as factors contributing to morphological alterations in the prostate [4].

Currently, two main classes of drugs are used to treat BPH: α1-adrenergic receptor antagonists such as doxazosin, terazosin, and tamsulosin, which ease lower urinary tract symptoms (LUTS) by relaxing the prostate and bladder muscles, and 5α-reductase inhibitors (5-αRi), such as finasteride or dutasteride, which inhibit the enzyme responsible for forming DHT, a key factor in BPH development. Despite the clinical benefit, conventional therapy of BPH is correlated with disturbances in sexual dynamics, significantly affecting men's quality of life [5].

Therefore, many patients initially prefer to use plant-based dietary supplements (DS) that contain sitosterol (SIT). SIT is a naturally occurring phytosterol, structurally related to cholesterol. It is currently used for its potential health benefits, including the reduction of total cholesterol levels and the risk of heart disease when included in a balanced diet [6]. The positive effects of SIT on BPH have been demonstrated in experimental animal studies, exhibiting anti-androgenic, pro-apoptotic, anti-inflammatory, and antioxidant effects. Although preclinical studies have demonstrated the beneficial effects of SIT in BPH, extrapolating these effects to humans is challenging [6-9].

SIT-based DS can be purchased from pharmacies, natural health stores, and websites. The quality of these DS is questionable especially because of the permissive legislation [10, 11]. The number of DS available on the market is continuously increasing, making it practically impossible to maintain consistent quality control.

The main objective of this study was to assess the concentration of SIT in DS available on the market by a simple and precise high-performance liquid chromatography (HPLC) method, to compare the actual concentrations with the ones declared on the label and to verify if this concentration correlated with the recommended dosage is sufficient for the beneficial effects in BPH.

For this purpose, we resorted to: (1) develop a method for extracting SIT from DS; (2) develop and validate an HPLC with UV detection method to quantify SIT; (3) qualitative and quantitative analysis of SIT in DS by the developed HPLC method.

## Materials and methods

Materials

All chemicals and reagents were of analytical grade purity and were purchased from various suppliers: methanol, acetonitrile, ethyl acetate, ethyl alcohol, potassium hydroxide, and dichloromethane were obtained from VWR International SAS (Fontenay-sous-Bois, France), diethyl ether from Lach-Ner (Czech Republic), hexane from ROTH (Karlsruhe, Germany), hydrochloric acid solution from Chemical Company (Iasi, Romania). The standards, SIT and cholesterol, were purchased from Thermo Fisher Scientific (Waltham, Massachusetts, USA). Ultra-pure water was obtained using a Milli-Q purification system (Merck Millipore Corporation, Burlington, MA, USA). The tested DS were purchased from pharmacies.

Chromatographic conditions

Chromatographic analysis was performed on a Merck Hitachi HPLC system consisting of L-7100 quaternary pump, L-7200 autosampler, L-7360 thermostat-controlled column, L-7455 DAD (Diode Array Detector), L-7000 interface, L-7612 solvent degasser, and D-7000 HSM-Manager software.

Separation was performed on a reversed-phase Gemini N-C18 chromatographic column with dimensions of 100 x 4.6 mm and particle size of 3 μm. The mobile phase used for separation had a composition of methanol (97%) and water (3%) in isocratic elution. The injection volume used was 20 µL. The flow rate was set at 1 mL/min with an analysis time of 12 min. The eluent was monitored using a DAD system, and the detection wavelength was set at 210 nm.

Calibration was performed on 15-400 μg/mL range, with nine levels of concentration, and each concentration was prepared and evaluated five times. Working solutions for the nine concentration levels were prepared by diluting the stock solution with methanol. Internal standard (ISTD) was cholesterol with a concentration of 1 mg/mL in methanol. The final concentration of ISTD in the standard solutions was 200 μg/mL.

The analytical method was validated for carry-over, selectivity, linearity, accuracy, precision within- and between-run, and yield of extraction. Five calibration sequences were tested during validation, each sequence containing a calibration curve with nine levels and appropriate four quality control samples with different concentrations including the lower limit of quantification and 80% of the upper limit of quantification. Quality control samples were obtained by diluting the stock solution with methanol.

Sample analysis

**Figure 1:**
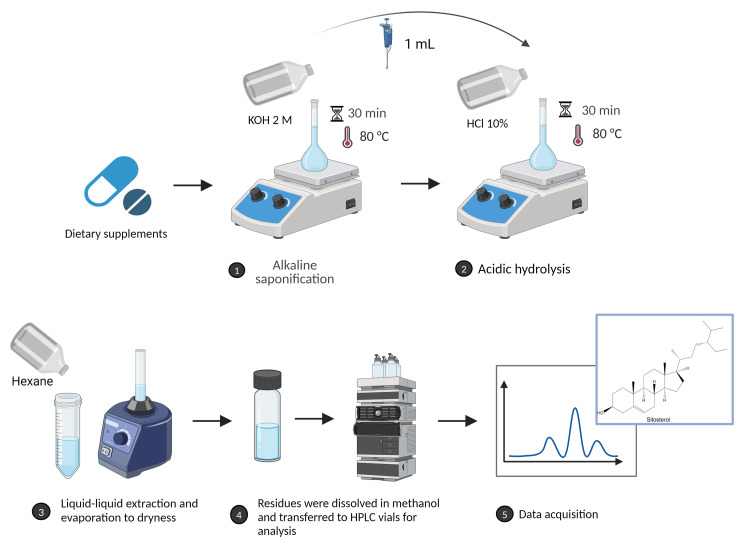
Schematic illustration of the workflow for SIT quantification in DS SIT: sitosterol; DS: dietary supplements

A number of five capsules of each DS were used and homogenized before extraction. The mass corresponding to one capsule, or an equal amount of the sample (0.5 g) was then weighted. If the total phytosterol content was higher than 2%, the amount taken for analysis was less than 0.5 g. For sample analysis, a modified Fibigr et al.'s method [12] was used. Thus, 0.5 g of DS sample was placed in a 25 mL flask, to which 15 mL of 2 M ethanolic KOH solution and 1 mL of 1 mg/mL ISTD solution were added. The mixture was then incubated for 30 min at 80°C with continuous stirring for saponification using a magnetic hotplate stirrer (DLAB MS-H280-Pro). The mixture was then cooled, and the flask was topped with ethanol. One milliliter of the obtained solution was pipetted into another 20 mL flask, to which 6 mL of diluted HCl (10%) was added for acidic hydrolysis. The mixture was then incubated for 30 min at 80°C with continuous stirring. The mixture was cooled, and 10 mL of hexane was added. The mixture was then vortexed (using a vortex mixer Grant-bio PV-1) for 1 min, the organic phase was removed, evaporated to dryness, and the residues were dissolved in methanol and transferred to HPLC vials for analysis. The workflow for SIT quantification in DS is described in Figure 1. Optimization of the extraction method of SIT from DS consisted of evaluating the importance of saponification, selection of the appropriate organic solvent for extraction, saponification time, and temperature applied to acidic hydrolysis.

## Results

Method validation

**Figure 2:**
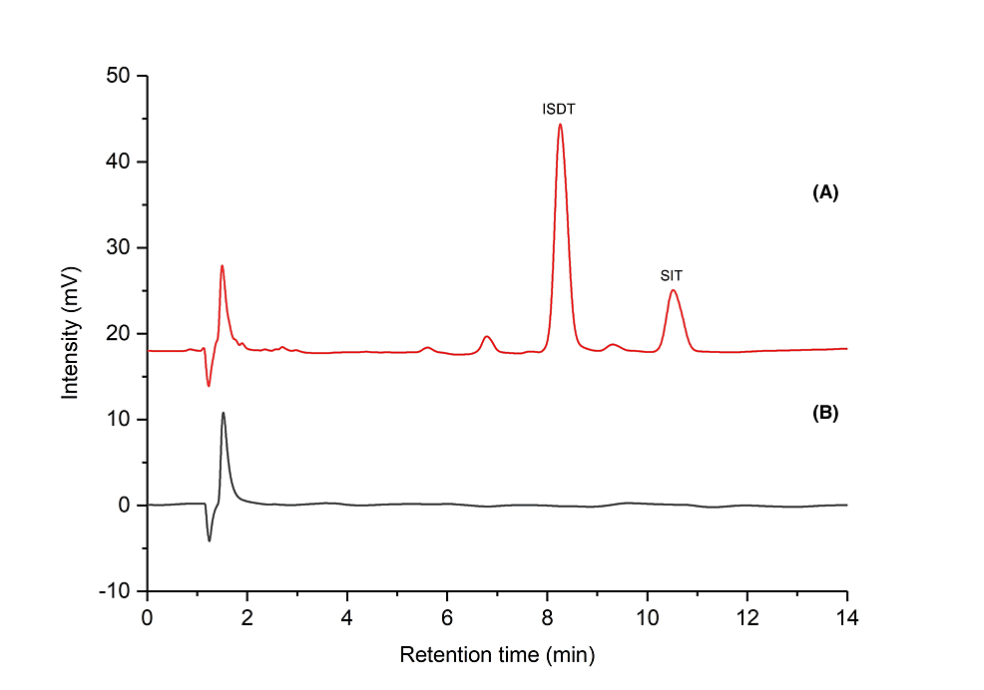
(A) Chromatogram of a SIT standard solution (RT = 8.26 ± 0.06 min for ISTD and RT = 10.54 ± 0.08 min for SIT); (B) Chromatogram of a blank solution RT: retention time; ISTD: internal standard; SIT: sitosterol

In the proposed chromatographic conditions, SIT and ISTD were separated at the baseline with a retention time (RT) of 10.54 ± 0.08 min for SIT and 8.26 ± 0.06 min for ISTD (Figure 2). The method selectivity was tested by comparing the chromatograms of placebo and lower limit of quantification (LLOQ) solutions, and no interferences were detected at the analyte retention time (10.54 min). Additionally, carry-over was evaluated by injecting a blank sample (mobile phase) immediately after a high-concentration standard solution (400 μg/mL), and no interferences were detected in the blank solution at the analyte retention time.

Each of the five calibration curves injected during the validation process was linear, with a coefficient of determination R2 > 0.99. Mean calibration curve was Area = 0.76±0.005 + 0.01±0.004, N = 9 levels, n = 5 replicates. Accuracy for the analyzed compound was between 98.53% and 101.03%, and the relative standard deviation (RSD%) ranged from 1.57% to 2.02% for within-day precision and from 1.68% to 2.11% for between-day precision (Table 1).

**Table 1:** The accuracy and precision of the SIT in the quality control samples C_STD _- concentration of the standard; C_ISTD _- concentration of the internal standard; SD - standard deviation; RSD - relative standard deviation

C_STD_/C_ISTD_	Intra-Day				Inter-Day			
	Mean	SD	RSD%	Accuracy%	Mean	SD	RSD%	Accuracy%
0.075	0.07	0.001	2.02	98.53	0.07	0.001	2.11	99.22
0.225	0.22	0.004	1.91	99.63	0.21	0.003	1.68	99.00
1	0.98	0.017	1.80	98.87	0.99	0.020	2.03	101.03
1.75	1.73	0.027	1.57	100.04	1.73	0.034	1.99	100.91

The yield of extraction by area ratio % of extracted and unextracted standard solutions was 89.87±1.67 %.

Optimal conditions for extraction

**Figure 3:**
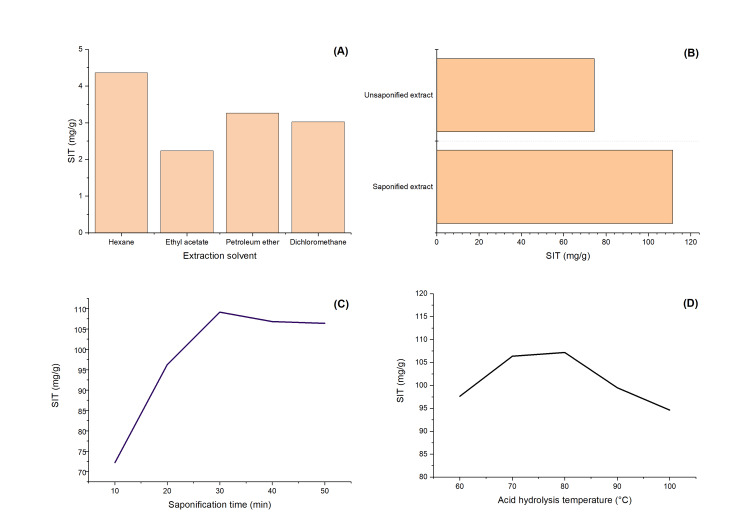
The influence of the (A) solvent extraction; (B) saponification process; (C) saponification time; and (D) acidic hydrolysis temperature in the extraction process of SIT from DS. (A) and (B) - optimized temperature of 80°C and time of 30 min; (C) and (D) - hexane as extraction solvent and full extraction process was applied

The optimization of extraction had as a starting point available literature data [12, 13]. The parameters that were varied were the temperature of acidic hydrolysis, time of saponification, and extraction solvent. As it can be seen in Figure 3, the highest yields of SIT were obtained by including the saponification process and using hexane as the extraction solvent at 80°C for 30 min.

Application of the developed method on a SIT-DS

**Figure 4:**
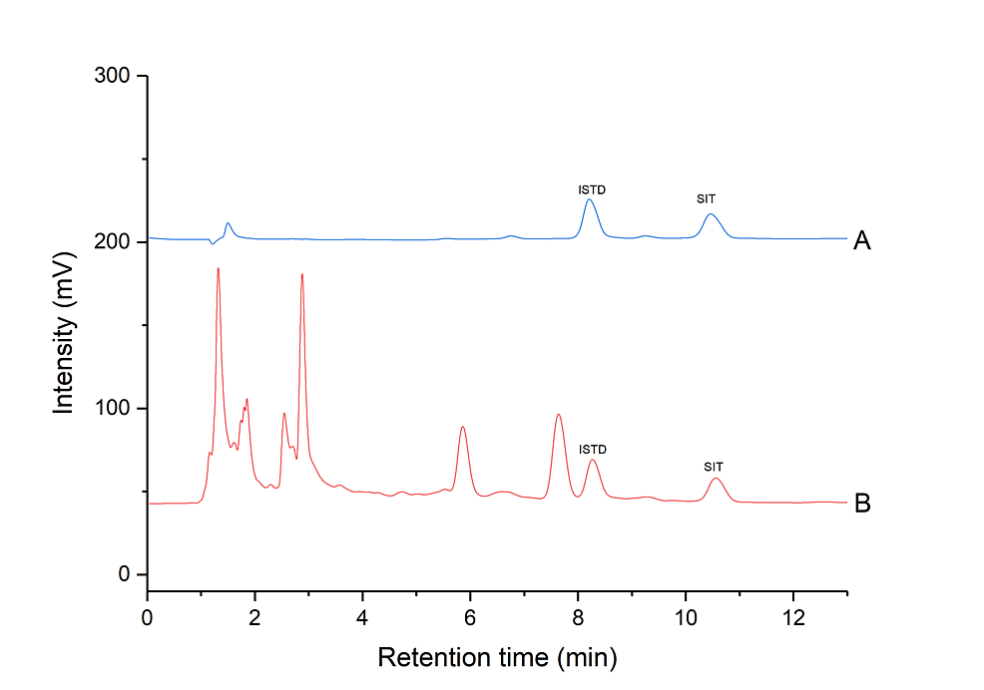
(A) Chromatogram of a SIT standard solution; (B) Chromatogram of an extracted DS containing SIT SIT: sitosterol; DS: dietary supplements; ISTD: internal standard

The developed and validated method was used to determine the SIT concentration in five different DS. Figure 4 shows the chromatogram of a DS in which the SIT has been identified. In DS_1_, DS_2_, and DS_3_, the declared amount by the manufacturer is a minimum of 0.32 mg SIT, while the measured amount is 2.98 ± 0.13, 4.71 ± 0.18, and 1.27 ± 0.01 mg, respectively. In DS_4_, the declared amount by the manufacturer is 130 mg, while the measured amount is 120.20 ± 0.13. In DS_5_, the declared amount by the manufacturer is 25 mg, while the measured amount is 10.67 ± 0.21. The results showed that the content of SIT measured in DS varied from 1.27 ± 0.01 to 120.20 ± 0.13 mg/unit. The results are presented in Table 2.

**Table 2:** SIT content in five different DS SD: standard deviation; SIT: sitosterol; DS: dietary supplements

DS	Recommended daily dose (unit)	Declared concentration of SIT (mg/unit)	Measured concentration (mg/unit) mean±SD	RSD%
DS_1_	1	minimum 0.32	2.98 ± 0.13	4.39
DS_2_	1	minimum 0.32	4.71 ± 0.18	3.84
DS_3_	1	minimum 0.32	1.27 ± 0.01	0.62
DS_4_	1	130	120.12 ± 0.63	0.52
DS_5_	1	25	10.67 ± 0.21	1.95

## Discussion

The present work analyzed the SIT content from five of the most well-marketed SIT-containing DS in Romania. The proposed chromatographic method proved linearity, accuracy, and precision in accordance with validation guidelines in pharmaceutical analysis and the extraction procedure is consistent and reproducible, HPLC being one of the analytical platforms considered as a reference in quality control of DS [14].

Our research results were consistent with findings reported in the literature, which confirmed that the SIT extraction process in this study was viable and efficient [6]. Comparing the concentration of SIT found in DS and the quantity declared by the manufacturer, one can seriously question if the available amount correlated with the recommended dosage is enough to trigger the beneficial effects of the DS in BPH.

A precise dose/kg bw of SIT in adults with BPH has not been established in any clinical protocols. However, based on several clinical studies, it is estimated that a dose of 60-140 mg of SIT alleviates the symptoms associated with BPH [6].

The Saw palmetto (Serenoa repens) extract represents the primary form in which SIT is found in the DS. The European Union (EU) legislation does not include specific regulations for SIT in DS. The United States Pharmacopoeia (USP) official in 2024 requires as a quality criterion that Saw palmetto extract contains no less than 0.2% total phytosterols out of which 0.1% SIT [15]. The same provisions were mentioned in the European Pharmacopoeia 11.5 (Ph. Eur 11.5) [16].

DS_1_, DS_2_, and DS_3_, according to the manufacturer label, should contain 320 mg of Saw palmetto extract and a minimum of 0.32 mg/unit SIT according to Pharmacopoeia provisions (no less than 0.1% SIT). Therefore, the content of SIT in all these three DS is according to the one declared on the label; SIT is present in a quantity higher than 0.32 mg/unit. Although SIT content in these DS is declared correctly by the manufacturer, according to literature data the dose is not sufficient to trigger beneficial effects. As a result, manufacturers are tending to prioritize the overall cumulative beneficial impact determined by the complex combination of compounds in the DS, rather than solely focusing on the effect of SIT.

Furthermore, the differences between the DS for different brands, almost 3.5 mg/unit as highlighted in our study, raise concerns regarding the quality and beneficial effects of the final product delivered to patients. This is especially concerning due to the discrepancies between the expected and actual doses of SIT per unit. This variability in content may be explained by the lack of a standardized extract of the active ingredient, the different industrial methods used to obtain the Saw palmetto extract, and last but not least the quality of the vegetal product. Other factors that could also contribute to the observed variation may include differences between batches and plant growth conditions [17].

The other two supplements (DS_4_ and DS_5_), according to the manufacturer, contained standardized extracts of Saw palmetto enriched with SIT. The data obtained showed that both DS contained a higher amount of SIT compared to extracts not enriched with SIT, but lower SIT compared to that reported by the manufacturer. DS_5_ has emerged as the sole supplement providing the necessary SIT quantity for effective BPH management, highlighting the importance of accurate labeling and quality assurance in DS.

Due to its increasing demand, limited cultivation area, and adverse environmental conditions that have negatively affected the availability of Saw palmetto berries in recent years, Saw palmetto extract has become relatively expensive, and thus, it is increasingly subjected to adulteration [18]. This issue has already been reported in several countries (India, China) [19, 20]. The reasons mentioned above could account for the relatively lower content compared to that reported on the label. In a study conducted by Kim et al., the SIT content in DS was compared to that in a standardized Saw palmetto extract. In contrast, the levels of plant sterols were 1.4-2 times higher than the reference values. According to the authors of the previously mentioned study, the products were adulterated, potentially by incorporating a lower amount of high-quality extract and supplementing with exogenous plant lipid fractions abundant in sterols [21].

## Conclusions

In conclusion, the investigation of SIT levels in DS aimed to shed light on their therapeutic efficacy for BPH by underscoring the challenges associated with the formulation of DS. The study findings emphasize the absence of standardization with regard to the active ingredients in DS. These ingredients are commonly listed as proprietary blends without specifying the exact nominal quantities. Manufacturers may prioritize the overall composition of supplements over the specific therapeutic effects of individual compounds, such as SIT. Furthermore, concerns about adulteration and variability in plant sources exacerbate this issue. As a result, this practice may impede the quantification of the beneficial effects post-administration and the execution of long-term efficacy and safety studies, particularly in the case of significant exposure in the elderly male population.

Furthermore, the study uncovered worries about insufficient oversight of the quality of DS containing SIT by manufacturers or government agencies. It emphasizes the necessity of conducting thorough testing to guarantee compliance with current regulations and to ensure that consumers benefit from high-quality and safe products. These results can be utilized as a starting point to introduce feasible analytical strategies for enhancing industry standards and implementing more rigorous quality control measures during the production and marketing of DS-containing SIT.
